# Effect of Exposure of Human Monocyte-Derived Macrophages to High, versus Normal, Glucose on Subsequent Lipid Accumulation from Glycated and Acetylated Low-Density Lipoproteins

**DOI:** 10.1155/2011/851280

**Published:** 2011-08-28

**Authors:** Fatemeh Moheimani, Joanne T. M. Tan, Bronwyn E. Brown, Alison K. Heather, David M. van Reyk, Michael J. Davies

**Affiliations:** ^1^Free Radical Group, The Heart Research Institute, 7 Eliza Street, Newtown, Sydney, NSW 2042, Australia; ^2^Faculty of Medicine, University of Sydney, Sydney, NSW 2006, Australia; ^3^Gene Regulation Group, The Heart Research Institute, Sydney, NSW 2042, Australia; ^4^School of Medical and Molecular Biosciences, University of Technology, Sydney, NSW 2007, Australia

## Abstract

During atherosclerosis monocyte-derived macrophages accumulate cholesteryl esters from low-density lipoproteins (LDLs) via lectin-like oxidised LDL receptor-1 (LOX-1) and class AI and AII (SR-AI, SR-AII) and class B (SR-BI, CD36) scavenger receptors. Here we examined the hypothesis that hyperglycaemia may modulate receptor expression and hence lipid accumulation in macrophages. Human monocytes were matured into macrophages in 30 versus 5 mM glucose and receptor expression and lipid accumulation quantified. High glucose elevated LOX1 mRNA, but decreased SR-AI, SR-BI, LDLR, and CD36 mRNA. SR-BI and CD36 protein levels were decreased. Normo- and hyperglycaemic cells accumulated cholesteryl esters from modified LDL to a greater extent than control LDL, but total and individual cholesteryl ester accumulation was not affected by glucose levels. It is concluded that, whilst macrophage scavenger receptor mRNA and protein levels can be modulated by high glucose, these are not key factors in lipid accumulation by human macrophages under the conditions examined.

## 1. Introduction

People with diabetes are at high risk of developing micro- and macrovascular diseases, with cardiovascular disease accounting for *ca*. 50% of deaths in these people [1]. Risk factors for this enhanced atherosclerosis include chronic elevated glucose (hyperglycaemia), insulin resistance, dyslipidaemias, and haemostatic abnormalities [2]. Intensive diabetes management can reduce cardiovascular events, with most of the decreased risk being related to lower HbA_1c_ levels [3], consistent with hyperglycaemia being a major contributing factor to diabetes-induced atherosclerosis. The mechanism(s) by which this occurs have not been fully elucidated [4]. Four principal processes have been proposed to explain the role of hyperglycaemia in the vascular complications of diabetes: increased polyol pathway flux; increased protein glycation and glycoxidation; activation of protein kinase C; increased hexosamine pathway flux [4]. 

Glycation involves the nonenzymatic reaction of glucose or reactive aldehydes (e.g., methylglyoxal, glyoxal, glycolaldehyde) with amine (e.g., *N*-terminal or Lys side chain), guanidine (Arg) and thiol (Cys) groups on proteins (e.g., the apoliprotein B-100 component of LDL) and yields multiple advanced glycation end products (AGEs) [5]. The rate and extent of reaction are dependent on the concentration and duration of exposure [5]. Reactive aldehydes, generated from glucose, amino acid and lipid oxidation, or metabolic pathways (e.g., via triosephosphates) [6], are present at elevated levels in the plasma of people with diabetes [7], and react with proteins much more rapidly than glucose [8, 9]. Free or protein-bound sugars also undergo autoxidation (autooxidative glycosylation or glycoxidation) to give radicals that can contribute to AGE generation. 

A key step in atherosclerosis is the uptake of modified low-density lipoproteins (LDLs) by vascular wall macrophages resulting in intracellular cholesterol accumulation and formation of lipid-laden cells [10]. Native LDL is internalised via LDL receptors in a tightly controlled feedback-regulated manner [10]. In contrast, LDL modified by multiple mechanisms, including oxidation, aggregation, and glycation, is taken up in an uncontrolled manner via multiple receptors, including lectin-like oxidised LDL receptor-1 (LOX-1), scavenger receptors class AI and AII (SR-AI and SR-AII), and class B scavenger receptors (SR-BI and CD36) [11, 12]. With regard to diabetes, glycation of LDL has therefore been proposed to enhance atherosclerosis through the promotion of lipid accumulation from LDL though this is not universally agreed upon (reviewed in [13]). LDL modification by methylglyoxal and glycolaldehyde *ex vivo*, under conditions that minimise oxidation, results in lipid accumulation by human monocyte-derived macrophages (HMDMs) and murine macrophages [14, 15], with this occurring primarily via SR-A and CD36 [15]. 

In addition to proatherogenic modifications to LDL, changes in the number of receptors for these modified particles, particularly the scavenger receptor types A and B, CD36, and LOX-1 which are not subject to feedback regulation, may also contribute to macrophage foam cell formation. ApoE knockout mice deficient in SR-A or CD36 have been reported to have similar levels of atherosclerosis to wild-type apoE knockout mice [16], whereas other studies have demonstrated that deletion of these receptors decreases atherosclerosis [17, 18]. Transgenic mice that overexpress SR-A, have provided evidence for augmented cellular cholesteryl ester accumulation from acetylated LDL [19]. In contrast, LDL receptor knockout mice overexpressing bovine SR-A, fed a “Western” diet, had reduced atherosclerosis [20], and similarly mice over-expressing human SR-A exhibited no changes in lesion area despite increased scavenger receptor activity *in vitro * [21]. These changes have however been reported principally in animal models, some of which did not have a background of diabetes, and in systems where the extent and type of lipoprotein modifications (both to low- and high-density lipoproteins) are likely to be highly heterogeneous and encompass both glycation and oxidation.

In the light of these conflicting data, this study examined whether maturation of human monocyte-derived macrophages (HMDMs) in high (30 mM) versus normal (5 mM) glucose results in elevated scavenger receptor mRNA and proteins levels and enhanced cellular cholesterol and cholesteryl ester accumulation from well-defined acetylated or glycated LDL particles.

## 2. Research Design and Methods

### 2.1. LDL Isolation, Modification, and Protein Quantification

LDL was isolated from multiple healthy, normoglycaemic, male and female donors who were not taking any medications or vitamin supplements. This investigation conforms with the principles outlined in the Declaration of Helsinki and was approved by the Ethics Review Committee (RPAH zone) of the Sydney South West Area Health Service (X05-0232, X09-0013). The LDL was glycated or acetylated as previously [9, 14]; these protocols do not give rise to significant oxidation of apoB protein, cholesterol, cholesteryl esters, or antioxidants as assessed by HPLC with UV, fluorescence, and electrochemical detection [9, 14]. Excess reagents were removed by elution through PD10 columns (Amersham) [9, 14]. LDL charge modification was quantified using 1% (w/v) agarose gels (Helena Laboratories) [14]. Protein concentrations were quantified using the Bicinchoninic Acid assay (Pierce) at 60°C, with BSA as standard [14]. Solutions were prepared using Nanopure water (Millipore-Waters) pretreated with washed Chelex-100 resin (Bio-Rad) to remove trace transition metal ions, with the exception of tissue culture reagents where sterile water (Baxter) was used.

### 2.2. Isolation and Culture of Human Monocyte-Derived Macrophages (HMDMs)

Monocytes were isolated from white blood cell concentrates provided by the Australian Red Cross Blood Service from healthy donors as described previously [15]. Cells (1 × 10^6^ in 1 mL X-VIVO 10 medium; BioWhittaker) were added to 12-well plates (Costar) and allowed to adhere (1-2 h). After washing the monocytes were incubated (5% CO_2_, 37°C) for 9–11 days in RPMI media (Sigma-Aldrich) containing 5 or 30 mM glucose, supplemented with 10% (v/v) heat-inactivated pooled human serum, 2 mM glutamine, and penicillin/streptomycin (100 U/mL and 100 *μ*g/mL, resp.; BioWhittaker). After 4 days, the medium was changed every 2 days. The resultant HMDMs were exposed to LDL (0 or 100 *μ*g/mL, 48 h) in RPMI media containing 10% (v/v) lipoprotein-deficient serum (in place of human serum) and either 5 or 30 mM glucose. Medium was subsequently collected and the cells lysed in water. Cell viability was assessed by lactate dehydrogenase (LDH) release [14]. Cellular cholesterol and cholesteryl esters were quantified, by mass, using HPLC [14]. Total cholesterol values were taken as the sum of free cholesterol and individual cholesteryl esters.

### 2.3. Assessment of HMDM Receptor Levels

Total RNA was isolated using TRI reagent (Sigma-Aldrich), and 100 ng total RNA was reverse-transcribed in a 10 *μ*L reaction using RNase H^+^ MMLV reverse transcriptase (Bio-Rad). The mRNA expression of the five genes of interest (SR-AI, SR-BI, LDLR, CD36, LOX1) and a housekeeping gene (**β**-actin) was determined by quantitative real-time PCR using iQ SYBR Green Supermix (Bio-Rad) and 20 pmol each of forward and reverse primer. The PCR conditions were as follows: 95°C for 3 mins, followed by 40 cycles of 95°C for 30 s, 56°C for 30 s, and 72°C for 30 s using the MyiQ Single-Color Real-Time PCR Detection System (Bio-Rad). The primer sequences were as follows: SR-AI (forward: 5′-TCACAATCAACAGGAGGACACT-3′ and reverse: 5′-CACGAGGAGGTAAAGGGCAAT-3′), SR-BI (forward: 5′-ACCGCACCTTCCAGTTCCAG-3′ and reverse: 5′-ATCACCGCCGCACCCAAG), LDLR (forward: 5′-CCCAGCGAAGATGCGAAGATAT-3′ and reverse: 5′-GGTGAAGAAGAGGTAGGCGATG-3′), CD36 (forward: 5′-TGCTGTCCTGGCTGTGTT-3′ and reverse: 5′-TGTTGCTGCTGTTCATCATCA-3′), LOX1 (forward: 5′-CTGGCTGCTGCCACTCTA-3′ and reverse: 5′-TTGCTTGCTCTTGTGTTAGGA-3′), and *β*-actin (forward: 5′-GAATTCTGGCCACGGCTGCTTCCAGCT-3′ and reverse: 5′-AAGCTTTTTCGTGGATGCCACAGGACT-3′). Relative changes in mRNA levels of the genes of interest were normalized using the ΔΔCT method to *β*-actin after using GENorm (http://medgen.ugent.be/~jvdesomp/genorm/) to assess that this was the most stable reference gene for this set of experiments. 

Protein was extracted from cells using RIPA buffer (150 mM NaCl, 1.0% w/v Igepal CA-630, 0.5% w/v deoxycholate, 0.1% w/v SDS, 50 mM Tris, pH 8.0; Sigma-Aldrich) and assayed for protein levels. Equal amounts of total cell lysate protein (100 *μ*g) were run on each gel. Samples were denatured by heating at 95°C for 5 mins with sample loading buffer containing *β*-mercaptoethanol. Denatured proteins were resolved by SDS-PAGE (10% w/v acrylamide) then transferred for 1 h at 13 V to polyvinyl difluoride membranes (Millipore), blocked using 5% (w/v) skim milk in TBST (50 mM Tris/HCl, 150 mM NaCl, 0.1 % v/v Tween 20; pH 7.5), washed three times with TBST, and incubated overnight with SR-AI antibody at 1 : 600 dilution (ab53566, Abcam), SR-BI antibody at 1 : 500 dilution (ab74413, Abcam), LDLR antibody at 1 : 200 dilution (ab30532, Abcam), LOX1 antibody at 1 : 500 dilution (ab60178, Abcam), or with *β*-actin antibody at 1 : 2000 dilution (MAB1501R, Chemicon). For the CD36 antibody, the blots were blocked using 3% (w/v) BSA in TBST overnight, followed by a 2-hour incubation at 21°C with CD36 antibody (ab36977, Abcam) at a dilution of 1 : 10,000. The membrane was then washed three times with TBST and incubated with the corresponding secondary antibody conjugated to horseradish peroxidase (HRP) at a 1 : 10,000 dilution for 2 h at 21°C. The membrane was again washed three times with TBST, and immunodetection was accomplished with enhanced chemiluminescence (Amersham Biosciences). Membranes were directly digitised (ChemiDoc XRS, Bio-Rad) and densitometric analysis completed using Quantity One Software (Bio-Rad). Normal glucose values were set as 100% to allow changes relative to this concentration to be determined.

### 2.4. Statistical Analyses

Data are mean + SEM from ≥3 separate experiments with triplicate samples, unless noted otherwise. Statistical analysis was performed with one-way analysis of variance (ANOVA) and Newman-Keul's *post hoc* analysis, except for the receptor levels that were analysed using a nonpaired, two-tailed Student's *t*-test. Significance was assumed if *P* < 0.05.

## 3. Results

### 3.1. Differentiation of Human Monocytes under Normal versus High Glucose Concentrations

Incubation of freshly elutriated human monocytes in 5 or 30 mM glucose for 9–11 days gave rise to macrophage cells (HMDMs) as reported previously [22]. The 30 mM glucose concentration did not give rise to morphological changes or significant differences in LDH release or protein levels (data not shown).

### 3.2. Effect of Normal versus High Glucose Concentrations on Receptor mRNA and Protein Levels

**Figure 1:**
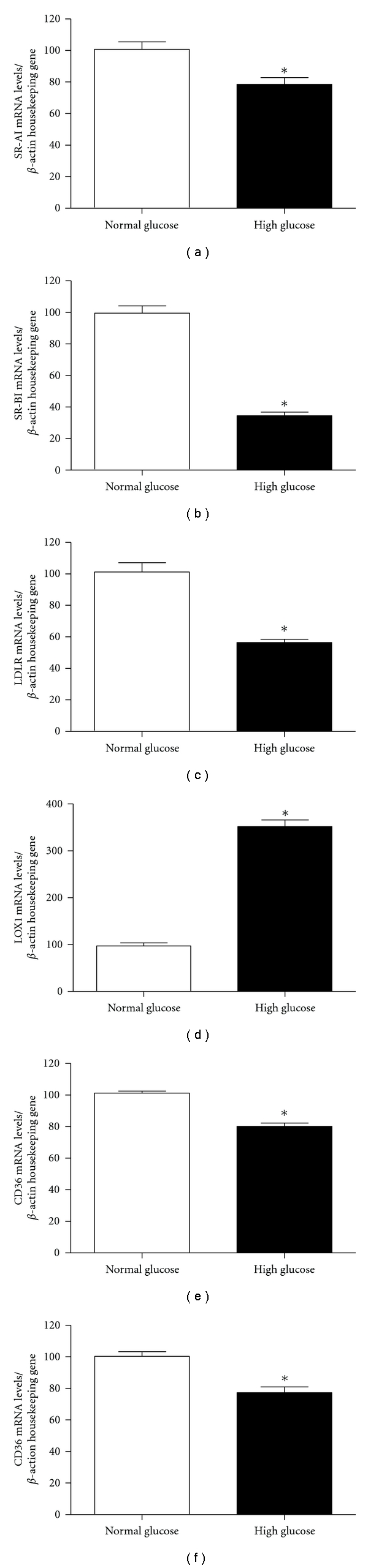
Gene expression data for (a) SR-AI, (b) SR- BI, (c) LDLR, (d) LOX1, and (e) CD36 from HMDM matured in either 5 mM (white bars) or 30 mM glucose (black bars) as measured by real-time quantitative PCR. Data (mean + SEM from *n* = 3 independent experiments) are expressed as a percentage change from the 5 mM concentration, which was set as 100%. *P < 0.05 for the 30 mM glucose concentration versus the 5 mM, using two-tailed unpaired *t*-tests.

**Figure 2:**
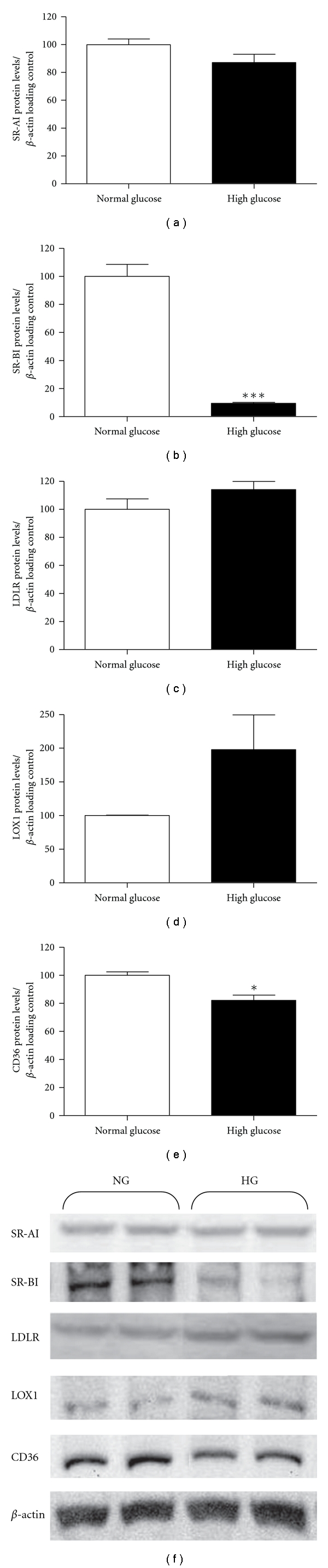
Protein levels (mean + SEM from *n* = 3 independent experiments) for (a) SR-AI, (b) SR-BI, (c) LDLR, (d) LOX1, and (e) CD36 from HMDM matured in either 5 mM (white bars) or 30 mM glucose (black bars), expressed as a percentage change from the 5 mM concentration (set as 100%). (f) Representative Western immunoblots (from three independent experiments) of 5 mM (NG) and 30 mM (HG) samples. *P < 0.05 for the 30 mM concentration versus the 5 mM using two-tailed unpaired *t*-tests.

Maturation of HMDM (in the absence of any exposure to LDL) in 30 mM compared to 5 mM glucose significantly decreased the expression (as assessed by real-time quantitative PCR) of SR-AI mRNA (79 + 4 versus 100 + 6%), SR-BI (42 + 2 versus 100 + 7%), LDLR (55 + 4 versus 100 + 6%), and CD36 (77 + 4% versus 100 + 3%) (Figure 1). In contrast, the expression of LOX1 was significantly increased (340 + 18% versus 101 + 5%) by 30 mM compared to 5 mM glucose (Figure 1). Receptor expression was also assessed at the protein level by Western blotting (Figure 2). Exposure to 30 mM glucose significantly decreased the protein expression of SR-BI (9 + 1% versus 100 + 8%) and CD36 (82 + 4% versus 100 + 3%) with a trend towards a decrease in SR-AI expression (87 + 6% versus 100 + 4%) when compared to the 5 mM glucose condition. In contrast, there was a trend towards an increase in the expression of both LDLR (114 + 6% versus 100 + 8%) and LOX1 (198 + 52% versus 100 + 1%) (Figure 2).

### 3.3. Generation of Acetylated and Glycated LDL

**Figure 3:**
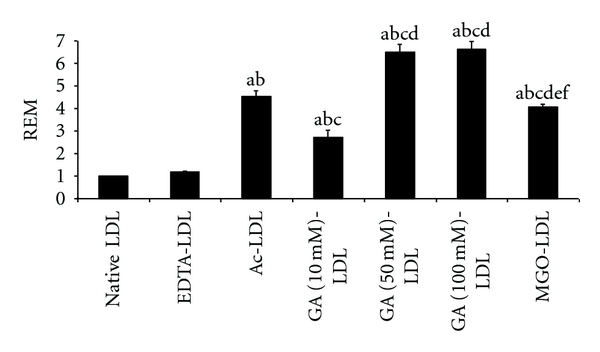
Relative electrophoretic mobility (REM) of unmodified (native) LDL (set as 1), LDL incubated with EDTA (EDTA-LDL), acetylated LDL (AcLDL), and LDL glycated by reactive aldehydes (glycolaldehyde, GA; methylglyoxal, MGO) at the indicated concentrations. Letters above bars indicate statistically significant differences at the *P* < 0.05 level for the following comparisons: “a” compared to native-LDL; “b” compared to EDTA-LDL; “c” compared to Ac-LDL; “d” compared to GA(10 mM)-LDL; “e” compared to GA(50 mM)-LDL; “f” compared to GA(100 mM)-LDL.

Acetylated (AcLDL) and glycated LDL (using methylglyoxal, MGO, or glycolaldehyde, GA) were generated from native LDL. The extent of modification to LDL induced by these protocols, including loss of Lys, Arg, and Trp residues, extent of aggregation, changes in mobility, the (minimal) extents of oxidation of apoB protein, cholesterol, cholesteryl esters, and consumption of antioxidants, has been reported previously [9, 14]. Modification was confirmed by quantification of the relative electrophoretic mobilities (REMs) of the treated particles compared to native LDL (nLDL; Figure 3). The AcLDL and glycated LDL preparations were statistically different to the incubation control (LDL incubated with EDTA), whilst the latter was not statistically different to nLDL. These data are in accord with our previously reported levels [9, 14]. The REMs of LDLs treated with 50 versus 100 mM glycolaldehyde were not statistically different.

### 3.4. Lipid Loading of HMDM Matured in Normal or High Glucose by Acetylated or Glycated LDL

**Figure 4:**
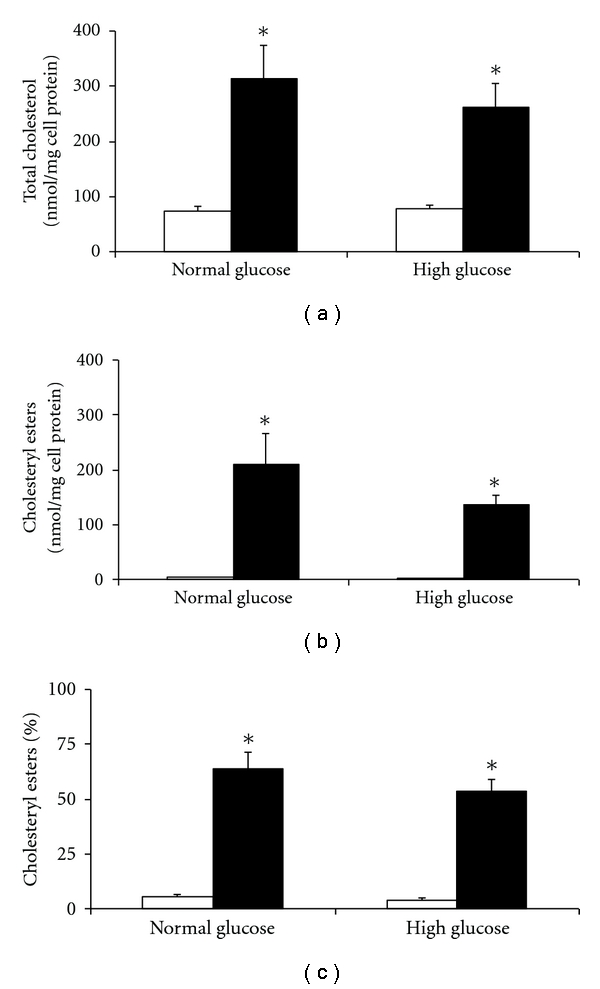
Effect of maturation in 5 versus 30 mM glucose on the accumulation of total cholesterol (a) and cholesteryl esters (b) and percentage cholesteryl esters (c), quantified by mass using HPLC, in HMDM cells after exposure to acetylated LDL (black bars) or control (no LDL, white bars). Data are mean ± SEM from four independent experiments. *P < 0.05, using one-way ANOVA and Newman-Keuls multiple comparison test. No differences between HMDM matured in normal and high glucose concentrations were observed for any of the data.

HMDMs, incubated in normal or high glucose, were exposed to AcLDL, highly glycated LDL, or control LDL (LDL incubated with EDTA to prevent oxidation) for 48 h. No differences were detected in cell morphology or number, LDH release or cell protein levels (data not shown). A significant increase in total cholesterol was detected for HMDM incubated with AcLDL for 48 h, compared to control cells not exposed to LDL (314 ± 60 versus 73 ± 9 nmol/mg cell protein for cells in 5 mM glucose; 263 ± 42 versus 77 ± 8 nmol/mg cell protein for cells in 30 mM glucose; Figure 4). Cholesterol accumulation from AcLDL for the 5 versus 30 mM glucose concentrations was not significantly different. Similar data were observed for cholesteryl esters when expressed as mass or as a percentage (AcLDL versus control: 210 ± 57 versus 5 ± 1 nmol/mg cell protein for 5 mM glucose; 136 ± 18 versus 3 ± 1 nmol/mg cell protein for 30 mM glucose; Figure 4), with no significant differences between the two glucose concentrations, for either AcLDL or control. There were no significant differences in the type or concentration of individual cholesteryl esters present in the cells (data not shown).

**Figure 5:**
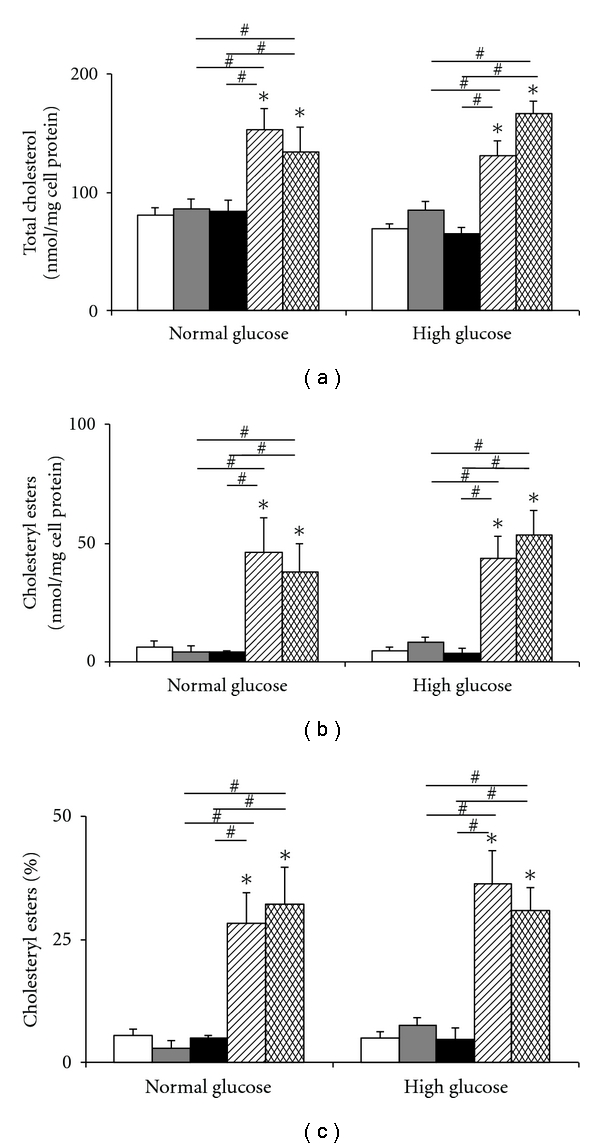
Effect of maturation in 5 versus 30 mM glucose on the accumulation of total cholesterol (a), cholesteryl esters (b) and percentage cholesteryl esters (c), quantified by mass using HPLC, in HMDM cells after exposure to native LDL (white bars), incubation control LDL (LDL incubated with EDTA: EDTA-LDL, gray bars), or modified LDL preglycated with 10 (black bars), 50 (striped bars) or 100 mM (hatched bars) glycolaldehyde. Data are mean ± SEM of three or more experiments. *P < 0.05, and ^#^ *P* < 0.05 as compared to other treatments, using one-way ANOVA and Newman-Keuls multiple comparison test.

 Incubation of either population of HMDM with LDL modified with 10 mM glycolaldehyde (GA(10 mM)-LDL) for 48 h did not have any significant effect on total cholesterol or cholesteryl ester mass or proportion of total cholesterol present as esters, compared to control cells. Incubation of HMDM with LDL preglycated with 50 or 100 mM glycolaldehyde resulted in a significant increase in cholesterol and cholesteryl ester levels compared to cells incubated with no LDL, control LDL (EDTA-LDL) or GA(10 mM)-LDL (Figure 5). There were no significant differences in the extent of cholesterol or cholesteryl ester accumulation between the 5 or 30 mM glucose conditions for the LDL glycated with 50 or 100 mM glycolaldehyde (Figure 5). There were no significant differences in the types or concentrations of cellular cholesteryl esters. 

HMDM incubated with LDL modified with 100 mM MGO, no LDL, or control LDL (EDTA-LDL) contained 109 ± 13, 76 ± 6, and 88 ± 2 nmol total cholesterol/mg cell protein, respectively, for the 5 mM glucose concentration, and 89 ± 13, 60 ± 8, and 80 ± 8 nmol total cholesterol/mg cell protein for the 30 mM glucose concentration, respectively. These values were not significantly different. Similarly, no statistical differences were observed for cellular cholesteryl ester mass and percentage of cholesterol esterification between the different conditions or for the types or concentrations of the individual cholesterol esters (data not shown).

## 4. Discussion

In this study the effect of maturation of HMDM in 30 versus 5 mM glucose on the accumulation of cholesterol and cholesteryl esters from well-characterized acetylated or highly glycated LDL has been examined. A glucose concentration of 30 mM was chosen as this reflects the limit detected in people with diabetes, compared to normal levels of ~5 mM. The cells were exposed to these glucose concentrations throughout the maturation period from monocytes to macrophages. Previous studies have shown that exposure of HMDM (and other macrophage cells) to glycated, but minimally oxidised, LDL results in a time- and concentration-dependent accumulation of cholesterol and cholesteryl esters [14, 15], and these data have been confirmed here. This accumulation, which occurs primarily via modified LDL uptake via SR-AI and CD36 [15], is dependent on the nature and extent of LDL modification. MGO and GA induced a more rapid and extensive modification of LDL than glucose and a subsequent greater lipid accumulation within the HMDM than glucose-modified LDL [14, 15, 23, 24]. 

High glucose concentrations have been reported to enhance the levels of CD36, SR-A, and LOX-1 receptors involved in LDL internalisation [25–27], and the role of these receptors in the development of atherosclerosis is well established [16–20, 28]. The majority of these studies have not however used well-characterised lipoprotein particles, so the role of LDL particle modification (and the extent of this process) in mediating lipid accumulation cannot be discerned. Contrary to previous findings, we have demonstrated that expression of these receptors was, with the exception of LOX-1, inhibited when human monocytes were differentiated under high glucose conditions. However, with the exception of CD36, the modulation of gene expression was not matched with significant changes in synthesis of the same receptors or lipid accumulation. These data indicate that there may be significant posttranslational controls on receptor protein concentrations and compensatory changes that offset the altered level of uptake mediators. This may include changes in receptor half-life and trafficking. 

Lipid accumulation by HMDM (which reflects the balance between multiple processes including uptake and efflux) has been quantified using known amounts of well-defined highly glycated (but not significantly oxidised) LDL particles. These particles were prepared using three methods which not only generated particles with markedly different patterns and extents of LDL alteration, but which also ensured particle homogeneity and minimised oxidation as a potential limiting and variable factor. Acetylation increases the negative charge of LDL particles via conversion of Lys residues to *N*-acetylated species with a corresponding loss of positive charge [29]. In contrast, glycolaldehyde targets Lys, Arg, and Trp residues, with Lys modified to a greater extent than Arg or Trp, while methylglyoxal preferentially modifies Arg residues [5, 14, 23, 30, 31]. The extent of change in particle charge (as judged by relative electrophoretic mobility) was comparable between acetylated and glycolaldehyde-modified LDL and greater than for methylglyoxal-modified LDL. In contrast to the major changes induced to LDL by these reactive aldehydes, previous studies have shown that exposure of LDL to high glucose concentrations (≤100 mM) for similar periods did not induce significant alterations (see, e.g., [9]).

The effects of different glycolaldehyde concentrations used in the LDL modification have been examined, to determine whether lipid accumulation is dependent on the degree of LDL modification. No significant differences were detected between the 30 and 5 mM glucose-exposed cells with any of these LDL preparations, though the extent of accumulation was less marked with LDL modified with 10, versus 50 or 100 mM glycolaldehyde. These data are consistent with the receptor level not being a critical factor in determining the extent of lipid accumulation. In contrast, the degree of particle modification is a major factor. Whether the rate of lipid accumulation is affected by altered receptor expression has not been examined in detail. However, exposure of macrophages to modified LDL *in vivo* is likely to occur throughout the lifetime of the monocyte/macrophage, so the kinetics of LDL uptake may not be the major determinant of the extent of loading relative to the impact of the nature and extent of LDL modification. 

These data appear to contradict a previous report where increased lipid loading was detected in cells exposed to high glucose [27]. However, it should be noted that different methods were employed to quantify lipid loading, with mass measurement used in the current study, and radiotracers in the previous study. The latter technique demonstrated a 40% increase in ^3^H-oleate incorporation into cholesteryl esters under hyperglycaemic conditions, rather than a net increase in total cholesterol mass. Previous studies have indicated that oleate incorporation into esters may not correlate directly with total cellular cholesterol accumulation in some macrophages [32]. Thus methodology and experimental design may account for this discrepancy. 

Glycolaldehyde and methylglyoxal were used to generate the glycated LDL, in place of glucose, as these species are much more reactive than glucose allowing for the generation of modified particles within a time frame consistent with the relatively short plasma half-life of LDL; glucose induces LDL modification at a much lower rate [9, 33]. Elevated, though variable, levels of reactive aldehydes, including methylglyoxal, have been detected in plasma from people with diabetes compared to healthy controls [7, 33, 34]. The high reactivity of these aldehydes [35] with Arg, Lys, His, and Cys side-chains and the *N*-terminal amino group, clearly limits the concentration of these materials in plasma. These reported values therefore represent steady-state concentrations, rather than the overall flux to which a protein may be exposed over its lifetime *in vivo*, given the short half-life (minutes) of reactive aldehydes in plasma [35]. The (high) bolus doses employed in the current study (and also by other groups; e.g., [36–39]) were chosen to model such lifetime exposure. Whether these steady-state plasma levels of aldehydes reflect those within cells is unclear, as no specific cellular measurements have been reported. Several processes have been identified, within the vessel wall, that have the potential to generate high levels of these aldehydes including intracellular production and decomposition of triose phosphates, glucose autooxidation [40], inflammation [41–43], and lipid oxidation [44]. These data suggest that higher levels of reactive aldehydes are likely to be present in atherosclerotic lesions, than in plasma, and potentially play a role in the macrovascular complications of diabetes.

This study has a number of limitations. Primary human monocyte-derived macrophages have been used in place of tissue macrophages due to the difficulty in obtaining the latter from donors. Furthermore, the cells were exposed to a constant glucose concentration, in contrast to the variable levels seen *in vivo*. The LDL used in this study was glycated *in vitro*, rather than using *in vivo* glycated materials, as this allows well-defined particles to be employed and at concentrations sufficient to ensure that the results were not confounded by the variable types and levels of modification observed *in vivo*. 

 In conclusion, this study suggests that changes to cellular receptor levels induced by chronic exposure of cells to high glucose do not significantly modulate cellular cholesterol and cholesteryl ester accumulation, or cholesterol ester type, by human macrophages. This was true despite significant changes in the expression of receptor mRNA and protein levels induced by high glucose concentrations. These data support hyperglycaemia-induced protein glycation (e.g., of lipoproteins) as being a major underlying cause for the increased incidence, and rate of development, of atherosclerosis in people with diabetes.

##  Conflict of Interests

The authors have no conflict of interests with regard to the studies reported in this paper.

##  Authors' Contribution

F. Moheimani carried out the cell and LDL experiments, carried out data analysis, and helped draft the manuscript. J. T. M. Tan carried out the gene expression/PCR experiments and carried out data analysis. B. E. Brown assisted with the cell and LDL experiments and data analysis. A. K. Heather designed and coordinated the gene expression/PCR experiments and contributed to the data analysis and manuscript preparation. D. M. van Reyk participated in the study design and helped draft the manuscript. M. Davies conceived the study, participated in the study design, and helped draft the manuscript. All authors read and approved the final paper.
